# One Cause of Chorea

**Published:** 1887-12

**Authors:** 


### *ONE CAUSE OF CHOREA.*

Dr. Wm. Osler, during the summer session of the Infirmary for Nervous Diseases, Philadelphia, delivered two lectures on the general etiology and symptoms of chorea. After enumerating a number of probable causes of this affection, he says, "that a really important factor is the strain of education, particularly in young girls during the third hemi-decade. Although, in only thirty cases, "over-work at school," "examinations," "worry about lessons," are given as exciting causes, I feel sure that, in a much larger proportion, the stimulation of school life is responsible for conditions which favor, if they do not actually precipitate, attacks. Keen, active-minded little girls, from ten to fourteen years of age, anxious to do well at lessons, stimulated in their efforts by parents and teachers, form a large contingent of cases. In London, Sturges has for some time studied this question with great care, and his papers on school-made chorea call attention to a serious danger in the forcing system, which prevails so largely in our modern methods of education."

